# Lens-induced myopization and intraocular pressure in young guinea pigs

**DOI:** 10.1186/s12886-020-01610-x

**Published:** 2020-08-25

**Authors:** Li Dong, Yi Fan Li, Hao Tian Wu, Hai Di Kou, Yin Jun Lan, Ya Xing Wang, Jost B. Jonas, Wen Bin Wei

**Affiliations:** 1 Beijing Tongren Eye Center, Beijing Key Laboratory of Intraocular Tumor Diagnosis and Treatment, Beijing Ophthalmology & Visual Sciences Key Lab, Medical Artificial Intelligence Research and Verification Laboratory of the Ministry of Industry and Information Technology, Beijing Tongren Hospital, Capital Medical University, 1 Dong Jiao Min Lane, Beijing, 100730 China; 2 Optometry Center, Beijing Tongren Hospital, Capital Medical University, Beijing, China; 3 Beijing Institute of Ophthalmology, Beijing Tongren Eye Center, Beijing Ophthalmology & Visual Science Key Lab, Beijing Tongren Hospital, Capital Medical University, Beijing, China; 4 Department of Ophthalmology, Medical Faculty Mannheim of the Ruprecht-Karls-University Heidelberg, Mannheim, Germany

**Keywords:** Intraocular pressure, Axial length, Myopia, Refractive error, Beta-blocker

## Abstract

**Background:**

Intraocular pressure (IOP) is an important physiological measure of the eye and is associated with some ocular disorders. We aimed to assess the influence of topical beta blocker-induced IOP reduction on lens-induced axial elongation in young guinea pigs.

**Methods:**

The experimental study included 20 pigmented guinea pigs (age: 2–3 weeks). Myopia was induced in the right eyes for 5 weeks with − 10 diopter lenses. The right eyes additionally received either one drop of carteolol 2% (study group, *n* = 10) or one drop of artificial tears daily (control group, *n* = 10), while the contralateral eyes of all animals remained untouched. The outcome parameter was axial elongation during the follow-up period. The mean of all IOP measurements taken during the study was referred to as mean IOP.

**Results:**

Greater axial elongation was associated with a shorter axial length at baseline (*P* < 0.001; standardized regression coefficient beta: − 0.54) and lens-induced myopization (*P* < 0.001; beta: 0.55). In the multivariable model, axial elongation was not significantly correlated with the IOP at study end (*P* = 0.59), the mean IOP during the study period (*P* = 0.12), the mean of all IOP measurements (*P* = 0.17), the difference between the IOP at study end and baseline IOP (*P* = 0.38), the difference between the mean IOP during the study period and the baseline IOP (*P* = 0.11), or the application of carteolol eye drops versus artificial tears eye drops (*P* = 0.07). The univariate analysis of the relationships between axial elongation and the IOP parameters yielded similar results. The inter-eye difference between the right eye and the left eye in axial elongation was significantly associated with the inter-eye difference in baseline axial length (*P* = 0.001; beta:-0.67) but not significantly correlated with the inter-eye difference in any of the IOP-related parameters (all *P* > 0.25).

**Conclusions:**

In young guinea pigs with or without lens-induced axial elongation, neither the physiological IOP nor the IOP reduced by carteolol, a topical beta-blocker, was associated with the magnitude of axial elongation. These results suggest that IOP, regardless of whether it is influenced by carteolol, does not play a major role in axial elongation in young guinea pigs.

## Background

Intraocular pressure (IOP) is an important part of the physiology and pathophysiology of the eye. A minimum IOP of approximately 2 to 5 mmHg is necessary for the eye to maintain a consistent form and shape and to be resistant to deformations induced by inner forces, such as caused by the ciliary muscle, and by external forces such as caused by the eyelids and extraocular muscles [1]. A minimum IOP of approximately 2 to 5 mmHg is also needed to prevent thickening of the choroidal compartment and to promote the orthograde axoplasmic flow in the retinal ganglion axons through the lamina cribrosa of the optic nerve head [2], to mention only few aspects. Axial myopia is characterized by the enlargement of the globe and can be differentiated between primary axial myopia and secondary axial myopia. In cases of primary axial myopia, mostly the posterior half of the globe enlarges. In cases of secondary myopia in eyes with congenital glaucoma, the eye wall in the anterior segment, including the cornea, and that in the posterior segment expands as the IOP increases [3–7]. Based on observations made in eyes with congenital glaucoma and in adult hypotonic phthisic eyes, the possibility that the IOP may also play a role in primary axial myopia has been discussed, and it has been debated whether IOP in the upper normal range contributes to the expansion and elongation of the globe [8–12]. The hypothesis is supported by findings reported in some population-based studies. In these investigations, a higher IOP was associated with a longer axial length and higher myopic refractive errors after the statistical model was adjusted for parameters influencing IOP [13, 14]. Similar results have been observed in hospital-based investigations [15–17]. However, other studies reported contradictory results: in the population-based investigations of the Central India Eye and Medical Study and the Beijing Eye Study, axial length was not significantly related to IOP [18, 19].

In view of the attention that myopia has received in recent decades and to obtain more information about the processes of emmetropization and myopization, we conducted this study to determine whether IOP and axial length are associated in young guinea pigs [20, 21].

## Methods

The experimental study included 20 pigmented guinea pigs (*Cavia porcellus*) with an age of 2–3 weeks and a body weight of 150–200 g at baseline. The Ethics Committee of the Beijing Tongren Hospital approved the study, and the ARVO Statement and the ARRIVE Guidelines for the use of animals in ophthalmic and vision research were taken into account. The animals were purchased from the Fang Yuanyuan farm in Beijing, China.

The guinea pigs were randomly divided into a study group (*n* = 10) and a control group (*n* = 10). For the guinea pigs in the study group, the right eyes underwent lens-induced myopization and additionally received one eye drop of carteolol hydrochloride 2% daily (Mikelan, Otsuka Pharmaceutical Co., Tokyo, Japan). For the guinea pigs in the control group, the right eyes also underwent lens-induced myopization and additionally received one eye drop of preservative free artificial tears daily (Hycosan, Ursapharm Arzneimittel GmbH, Saarbrücken, Germany). The topical application of the drug or artificial tears was performed daily at 3 pm. The left eyes in both groups did not undergo any interventions, nor did they receive any eye drops.

To induce myopization, we glued goggles (refractive power: − 10 diopters; diameter: 15 mm, optical zone: 12 mm) at the start of the study onto the skin of the orbital rim of the right eyes after the skin had been cleaned and shaved. The goggles were removed daily to administer the eye drops and to perform weekly examinations. The examinations included tonometry (Tono-Pen, Reichert Inc., NY, USA) under topical anesthesia (oxybuprocaine hydrochloride eye drops; Santen Co., Osaka, Japan), followed by refractometry (streak retinoscopy, 66 vision tech Co., Jiangsu, China) under cycloplegia (using one drop of tropicamide), and ocular biometry (A/B-mode scan ultrasonography, oscillator frequency: 11 MHz, Quantel Co., Les Ulis, France). The axial length was defined as the distance from the anterior border of the cornea to the inner surface of the retina. The animals were awake during the examinations, and lid retractors were not used. The IOP was measured at 4 pm on each examination day. For each measure, three IOP measurements were taken, and the mean value was used for further statistical analysis. Care was taken that the guinea pigs could open their eyes and blink freely under the goggles. The goggles were examined daily to ensure they were clean and placed appropriately; otherwise, they were detached, cleaned, and reattached. The application of the eye drops and the application of the goggles were started on the same day.

All guinea pigs remained in an environment at a constant temperature of 26 °C. The circadian day/night rhythm was set to be 12 h/12 h (the lighting automatically changed at 8 am and 8 pm) with a luminous intensity of 500 lx. After the study, all animals were sacrificed by an intraperitoneal injection of pentobarbital sodium with an overdose of 600 mg per kg weight.

For statistical analysis, we used a commercially available statistical analysis program (SPSS, version 25.0, IBM-SPSS, Chicago, IL, USA). We first calculated the mean and standard deviation (SD) of axial length, refractive error and IOP. We then compared the parameters between the left and right eyes using Student’s t-test for paired samples and we compared the parameters of the two different groups using Student’s t-test for unpaired samples. To assess the relationships between the variables, we first performed a univariate analysis followed by a multivariable analysis (defined as a statistical model with multiple independent or response variables). We calculated the standardized regression coefficient beta, the non-standardized regression coefficient B, and its 95% confidence interval (CI). A *P*-value was considered to indicate statistical significance if it was smaller than 0.05.

## Results

Immediately before treatment, the IOP, refractive error and axial length did not differ significantly between the study group and the control group in terms of the right eyes only or the left eyes only, and they did not significantly differ between the right eyes and left eyes in general (all *P* > 0.20) (Tables 1, 2). For the whole study population, the mean IOP at baseline was 15.0 ± 3.0 mmHg, the mean axial length was 7.98 ± 0.05 mm, and the mean refractive error was + 1.89 ± 0.66 diopters (Tables 1 & 2).

**Table 1:** Measurements (mean ± standard deviation) of guinea pigs at baseline and at study end

	Study group, right eyes	Control group, right eyes	*P*-Value	Study group, Left eyes (no intervention)	Control group, left eyes (no intervention)	*P*-value
Baseline						
IOP (mmHg)	14.3 ± 2.6	15.0 ± 2.1	0.52	15.4 ± 3.4	15.4 ± 3.9	1.00
RE (D)	1.83 ± 0.88	1.93 ± 0.66	0.78	1.75 ± 0.55	2.08 ± 0.55	0.21
AL (mm)	7.99 ± 0.04	7.98 ± 0.07	0.61	7.98 ± 0.05	7.97 ± 0.05	0.76
Study end						
IOP (mmHg)	12.2 ± 2.4	18.8 ± 3.6	< 0.001	15.0 ± 3.3	16.0 ± 3.9	0.54
RE (D)	−1.65 ± 1.80	−3.48 ± 2.06	0.049	− 0.93 ± 2.05	− 0.18 ± 1.08	0.32
Change in RE (D)	−3.48 ± 1.75	−5.40 ± 2.38	0.055	−2.68 ± 2.54	−2.25 ± 1.16	0.64
AL (mm)	8.61 ± 0.12	8.70 ± 0.06	0.038	8.51 ± 0.05	8.53 ± 0.10	0.70
Axial elongation (mm)	0.62 ± 0.15	0.73 ± 0.09	0.07	0.53 ± 0.06	0.55 ± 0.12	0.62

*IOP* Intraocular pressure, *RE* Refractive error, *D* Diopter, *AL* Axial length. The *P*-values indicate the statistical significance of the difference between the two groups in the preceding two columns of the table

**Table 2:** Measurements (mean ± standard deviation) of guinea pigs at baseline and at study end

	Study group, right eyes	Study group, left eyes	*P*-Value	Control group, right eyes	Control group, left eyes	*P*-Value
IOP (mmHg)						
Baseline	14.3 ± 2.6	15.4 ± 3.4	0.43	15.0 ± 2.1	15.4 ± 3.9	0.78
1 week	11.2 ± 1.9	16.4 ± 4.0	0.002	18.6 ± 4.0	15.2 ± 3.3	0.05
2 weeks	11.4 ± 1.9	17.1 ± 3.6	0.001	16.9 ± 5.0	14.5 ± 4.0	0.25
3 weeks	11.2 ± 1.5	17.0 ± 3.6	0.000	17.7 ± 4.7	16.3 ± 4.3	0.49
4 weeks	11.8 ± 1.8	15.3 ± 3.6	0.01	18.2 ± 4.4	15.0 ± 3.4	0.09
5 weeks	12.2 ± 2.4	15.0 ± 3.3	0.045	18.8 ± 3.6	16.0 ± 3.9	0.11
RE (D)						
Baseline	1.83 ± 0.88	1.75 ± 0.55	0.82	1.93 ± 0.66	2.08 ± 0.55	0.59
1 week	0.98 ± 1.30	1.45 ± 0.63	0.31	1.23 ± 0.74	1.33 ± 0.41	0.71
2 weeks	0.18 ± 1.09	1.35 ± 0.41	0.008	0.35 ± 1.52	1.28 ± 0.43	0.09
3 weeks	− 0.38 ± 1.08	0.23 ± 1.30	0.28	− 1.60 ± 1.41	0.78 ± 0.85	< 0.001
4 weeks	−1.10 ± 1.53	− 0.40 ± 1.58	0.33	−2.48 ± 1.72	0.33 ± 1.02	0.001
5 weeks	−1.65 ± 1.80	− 0.93 ± 2.05	0.41	− 3.48 ± 2.06	−0.18 ± 1.08	0.001
AL (mm)						
Baseline	7.99 ± 0.04	7.98 ± 0.05	0.63	7.98 ± 0.07	7.97 ± 0.05	0.89
1 week	8.08 ± 0.05	8.06 ± 0.04	0.28	8.09 ± 0.06	8.05 ± 0.05	0.18
2 weeks	8.20 ± 0.10	8.18 ± 0.05	0.68	8.26 ± 0.09	8.17 ± 0.06	0.02
3 weeks	8.38 ± 0.06	8.29 ± 0.06	0.005	8.42 ± 0.07	8.32 ± 0.10	0.02
4 weeks	8.48 ± 0.09	8.42 ± 0.06	0.09	8.55 ± 0.08	8.44 ± 0.08	0.007
5 weeks	8.61 ± 0.12	8.51 ± 0.05	0.03	8.70 ± 0.06	8.53 ± 0.10	0.000

*IOP* Intraocular pressure, *RE* Refractive error, *D* Diopters, *AL* Axial length. The *P*-values indicate the statistical significance of the difference between the two groups in the preceding two columns of the table

At the end of the study, the IOP was significantly (*P* < 0.001) lower in the right eyes of the study group, in which carteolol was applied, than in the right eyes of the control group, in which artificial tears were applied (Table 1). Correspondingly, within the study group, the IOP was lower (*P* = 0.035) at the end of the study than at baseline in the eyes in which carteolol was applied, while the IOP at study end did not differ significantly from the IOP at baseline in the eyes in which artificial tears were applied (*P* = 0.79). Within the control group, the right eyes, in which artificial tears were applied and which underwent lens-induced myopization, had a significantly (*P* = 0.007) higher IOP at study end than at baseline, while the IOP in the left eyes, in which eye drops were not applied and which did not undergo lens-induced myopization, did not differ significantly between baseline and study end (*P* = 0.79) (Table 1).

At study end, the refractive error was significantly (*P* < 0.001) more negative (more myopic), and the axial length was significantly (*P* < 0.001) longer than at baseline for all groups in the study population, including the left eyes without lens-induced myopization. At study end, the axial length was significantly shorter in the right eyes of the study group (carteolol application and lens-induced myopization) than in the right eyes of the control group (artificial tears application and lens-induced myopization) (8.61 ± 0.12 mm versus 8.70 ± 0.06 mm; *P* = 0.038). The change in axial length from baseline to study end (magnitude of axial elongation) did not differ significantly between both groups (0.62 ± 0.15 mm versus 0.73 ± 0.09 mm; *P* = 0.07). Similarly, the refractive error at study end was significantly less negative (less myopic) in the right eyes of the study group (carteolol application and lens-induced myopization) than in the right eyes of the control group (− 1.65 ± 1.80 diopters versus − 3.48 ± 2.06 diopters; *P* = 0.049), while the change in refractive error did not differ significantly (− 3.48 ± 1.75 diopters versus − 5.40 ± 2.38 diopters; *P* = 0.055) between the two groups.

**Fig. 1:**
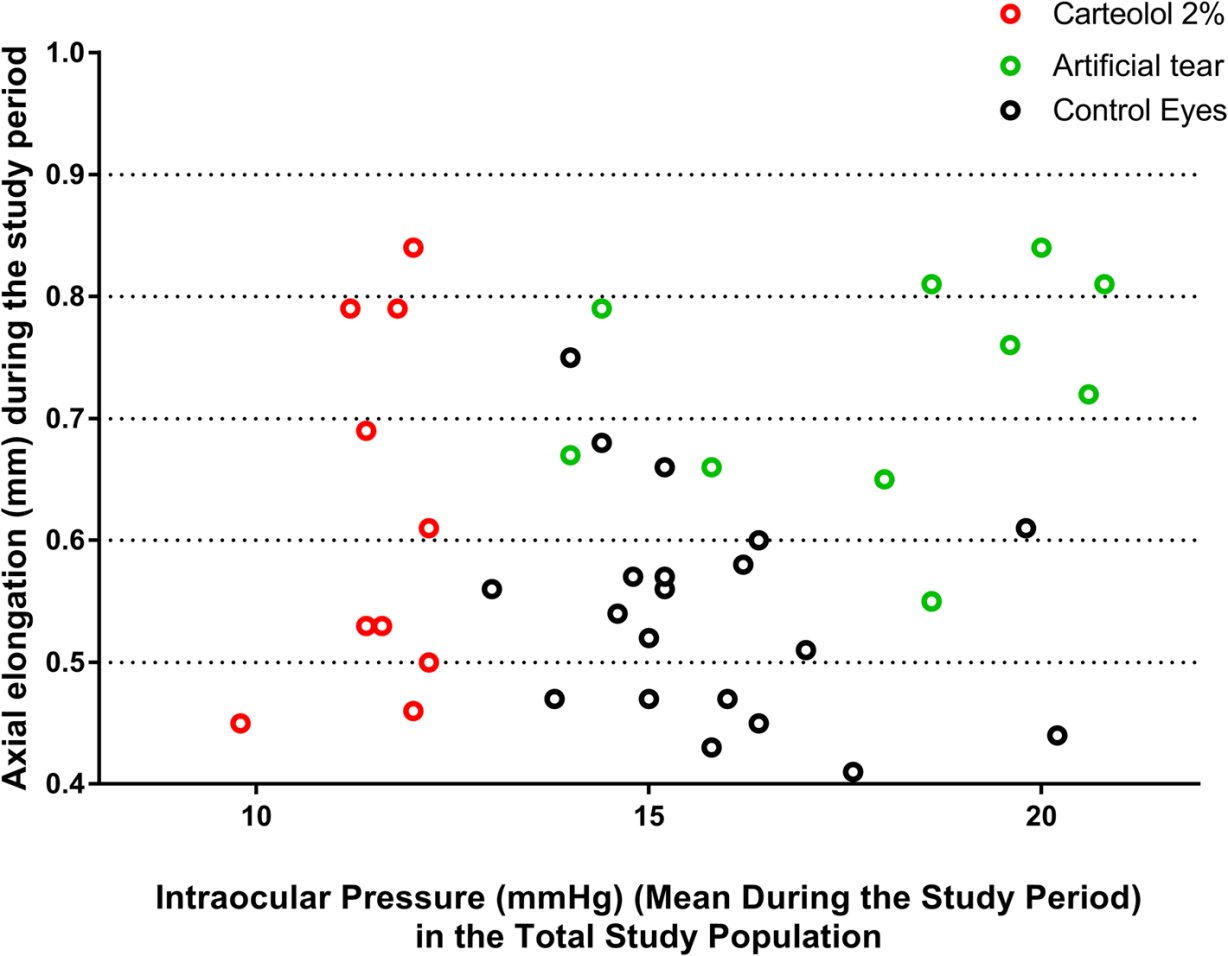
Scattergram showing the association between the mean intraocular pressure during the study period (measured at weekly intervals) and axial elongation (the final axial length minus the initial axial length) in the total study population. The treated eyes correspond to the right eyes, which underwent lens-induced myopia and received either carteolol 2% (red circles) or artificial tears (green circles); the control eyes correspond to the left eyes, which did not undergo any interventions or therapies (black circles)

**Fig. 2:**
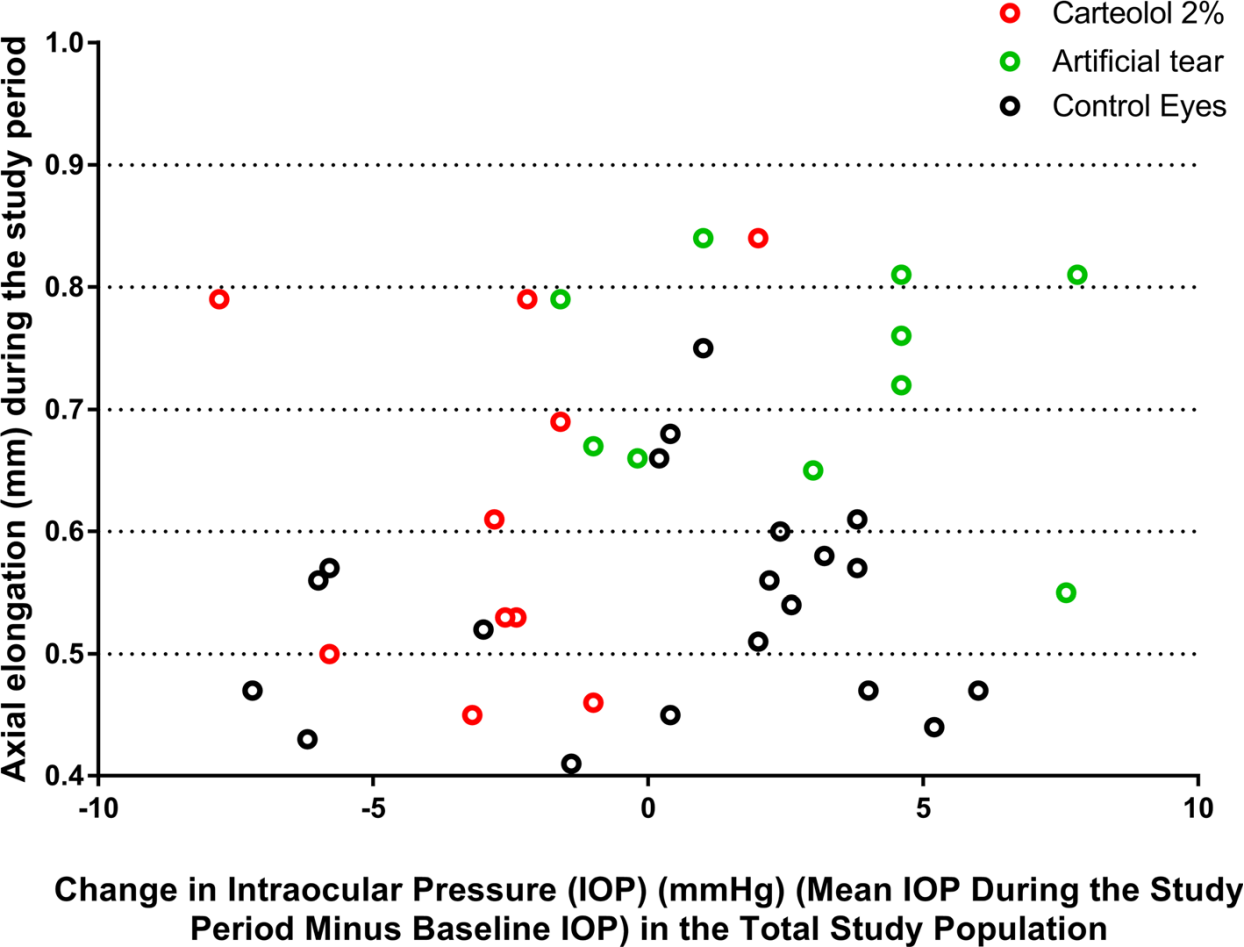
Scattergram showing the association between the change in intraocular pressure during the study period (at weekly intervals) and axial elongation (the final axial length minus the initial axial length) in the total study population. The treated eyes correspond to the right eyes, which underwent lens-induced myopia and received either carteolol 2% (red circles) or artificial tears (green circles); the control eyes correspond to the left eyes, which did not undergo any interventions or therapies (black circles)

Taking into consideration the whole study population and both eyes of each animal, greater axial elongation was associated with a shorter axial length at baseline (*P* < 0.001; beta: − 0.51). Axial elongation was more pronounced in the right eyes than in the left eyes (*P* < 0.001) (univariate analysis). The magnitude of axial elongation was not significantly correlated with the IOP at baseline (*P* = 0.48), the mean IOP during the study period (*P* = 0.41) (Fig. 1), the difference in IOP at study end and at baseline (*P* = 0.53), the difference between the mean IOP during the study period and the IOP at baseline (*P* = 0.26) (Fig. 2), or the mean of all IOP measurements (*P* = 0.51) (univariate analysis). When only the right eyes were taken into account and when the study population was subdivided into the study group (carteolol application) and the control group (artificial tears application), similar results were observed.

The multivariable analysis included the magnitude of axial elongation as the dependent variable and axial length at baseline and right eye versus left eye (i.e., lens-induced myopization versus no lens-induced myopization) as independent variables. Both independent variables remained to be significantly (*P* < 0.001) associated with axial elongation (Table 3). When IOP-related parameters were included as independent variables in that model, they were not found to be significantly associated with the magnitude of axial elongation during the study period. The IOP-related parameters included the IOP at study end (*P* = 0.59), the mean IOP during the study period (*P* = 0.12), the mean of all IOP measurements (*P* = 0.17), the difference between the IOP at study end and the baseline IOP (*P* = 0.38), and the difference between the mean IOP during the study period and the baseline IOP (*P* = 0.11). Similarly, when the group parameter for the study group (carteolol application) and the control group (artificial tears application) was included as an independent variable, it was not significantly (*P* = 0.07) correlated with axial elongation.

**Table 3:** Multivariable analysis of the associations of the amount of axial elongation during the study period in young guinea pigs

	*P*-value	Standardized regression coefficient beta	Non-standardized regression coefficient B	95% confidence interval	Variance inflation factor
AL at baseline	< 0.001	− 0.54	− 1.35	− 1.91, − 0.79	1.01
Lens-induced axial elongation (right eyes) versus no lens-induced axial elongation (left eyes)	< 0.001	− 0.55	− 0.14	− 0.20, − 0.08	1.01
Parameters added separately to the model					
IOP at study end	0.59	0.06	0.002	−0.005, 0.009	1.00
Mean IOP except for baseline value	0.12	0.17	0.008	−0.002, 0.017	1.04
Mean of all IOP	0.17	0.16	0.008	−0.004, 0.019	1.05
IOP at study end minus the baseline IOP	0.38	0.10	0.003	−0.004, 0.009	1.01
Mean IOP during the study period minus baseline IOP	0.11	0.18	0.006	−0.001, 0.013	1.00
Study group versus control group	0.07	0.20	0.05	−0.005, 0.11	1.01

*AL* axial length, *IOP* Intraocular pressure

In univariate analysis, the inter-eye difference in axial elongation between the right eyes and the left eyes was significantly associated with the inter-eye difference in baseline axial length (*P* = 0.001; beta: -0.67; B: -1.57; 95%CI: − 2.43, − 0.71) but not significantly correlated with the inter-eye difference in any of the IOP-related parameters (difference between the IOP at study end and IOP at baseline: *P* = 0.19; difference between the mean of all IOP measurements during the study period and baseline IOP: *P* = 0.09; mean of all IOP measurements: *P* = 0.25; mean of all IOP measurements during the study period: *P* = 0.19). Correspondingly, when any IOP-related parameter was included in the model of the association between inter-eye differences in axial elongation and inter-eye differences in baseline axial length, it did not reveal any other significant associations (difference between IOP at study end and IOP at baseline: *P* = 0.35; difference between the mean of all IOP measurements during the study period and baseline IOP: *P* = 0.25; mean of all IOP measurements: *P* = 0.27; mean of all IOP measurements during the study period: *P* = 0.25).

Similarly, the difference between the right eyes and the left eyes in the change in refractive error during the study period was not significantly related to any of the IOP-related parameters (all *P* ≥ 0.19).

## Discussion

In this experimental study in young guinea pigs, greater axial elongation was associated with a shorter axial length at baseline (*P* < 0.001) and with lens-induced myopization (*P* < 0.001), while it was not significantly correlated with the IOP at study end, mean IOP during the study period, difference in IOP at study end and at baseline, difference between the mean IOP during the study period and the baseline IOP, or the study group (carteolol application) versus control group (artificial tears application). In a similar manner, the inter-eye difference between the right eye and the left eye in axial elongation was significantly associated with the inter-eye difference in baseline axial length (*P* = 0.001) but not with the inter-eye difference in any of the IOP-related parameters. In young guinea pigs with or without lens-induced axial elongation, neither the physiological IOP measurements nor the reduced IOP measurements induced by a topical beta-blocker were associated with the magnitude of axial elongation.

The results of our study agree with the findings shown in previous investigations, some of which used the form-deprived myopic chick model (Table 4). In the study by Schmidt and colleagues, reduction of IOP by topical application of the beta-blocker timolol was not markedly effective against the development of myopia in chicks [9]. Similarly, our study showed that the topical application of the beta-blocker carteolol was not associated with axial elongation in young guinea pigs. Correspondingly, a clinical trial that compared children who received 0.25% timolol eye drops and children who used bifocal spectacles showed that these interventions did not significantly affect the progression of myopia [23]. Additionally, other clinical studies showed that topically applied beta-blockers did not affect the rate of myopia progression [24, 25]. Jin and Stjernschantz examined the influence of prostaglandins on the development of myopia in chicks and found that indomethacin administered intramuscularly, subconjunctivally or intravitreally as well as exogenous prostaglandin PGE2, prostaglandin PGF2alpha and latanoprost acid administered subconjunctivally or topically, did not have a significant effect on myopia development [10]. Interestingly, the intravitreal application of PGF2alpha significantly attenuated myopia development. Moreover, Jin and Stjernschantz reported that prostaglandins presumably lowered the IOP; however, they did not measure the IOP.

**Table 4:** A review of animal studies reporting the effects of IOP-lowering drugs on myopia progression

Authors	Species	Myopia	Method of tonometry	α- agonist	β-blocker	Proteinoid	IOP	Myopia progression
Schmid et al. [9]	Chick	FDM, LIM	Applanation tonometer	–	Timolol	–	Decrease	NE
Jin et al. [10]	Chick	FDM	No report	–	–	Latanoprost	No report	NE
Carr et al. [22]	Chick	FDM	No report	Brimonidine	–	–	No report	Decrease
Liu et al. [11]	Guinea pig	LIM	Rebound tonometry	Brimonidine	–	–	Decrease	Decrease
El-Nimri et al. [12]	Guinea pig	FDM	Rebound tonometry	–	–	Latanoprost	Decrease	Decrease
Dong et al. [This study]	Guinea pig	LIM	Applanation tonometry	–	Carteolol	–	Decrease	NE

*IOP* Intraocular pressure, *FDM* Form deprivation myopia, *LIM* Lens-induced myopia, *PG* Prostaglandin, *NE* No effect

The observations made in these investigations, including our study, are inconsistent with the results of the recent study by El-Nimri and Wildsoet conducted in two-week-old pigmented guinea pigs with unilateral form deprivation myopia; compared with the control group, that received artificial eye drops, the study group, that received latanoprost eye drops, showed a significant reduction in IOP and axial elongation [12]. At the 9-week follow-up, compared to the contralateral eyes, the eyes that received latanoprost eye drops showed significantly lower IOP readings, and the intereye difference was significantly larger in the study group, which unilaterally received latanoprost eye drops, than in the control, which unilaterally received artificial eye drops (− 5.17 ± 0.96 mmHg versus 1.80 ± 1.16 mmHg; *P* < 0.001). The interocular differences in axial length did not change significantly from baseline to the study end in the study group (from 0.02 ± 0.02 to 0.06 ± 0.02 mm; *P* = 0.20), while the interocular differences in axial length had significantly increased at the study end in the control group (from 0.00 ± 0.02 to 0.29 ± 0.04 mm; *P* < 0.001). Correspondingly, the inter-eye difference in refractive error slightly increased in the study group (from − 0.15 ± 0.35 diopters to − 2.25 ± 0.54 diopters; *P* = 0.03) and markedly increased in the control group (from + 0.03 ± 0.36 diopters to − 8.2 ± 0.71 diopters; *P* < 0.001). These discrepancies in the results of the studies may be explained by the differences between beta-blockers and prostaglandin derivatives as well as differences in the species studied, as the chick sclera has an inner cartilage layer in addition to the fibrous layer; thus, the mechanisms of globe elongation may differ between chicks and guinea pigs.

In another study in guinea pigs, the reduction of IOP by topical application of the α-adrenergic agonist brimonidine was also associated with a reduction in the rate of progression of myopia [11]. In that study, Liu and colleagues studied the right eyes of 36 guinea pigs, in which myopia was induced by goggles, as in our study. In the study groups that topically received 0.1% brimonidine eye drops only or 0.2% brimonidine eye drops only, the refractive error was less myopic (*P* = 0.024 and *P* = 0.006, respectively), and the axial length was shorter (*P* = 0.005 and *P* = 0.0017, respectively) than in the control group. Similarly, the combination of brimonidine 1% or 2% with 2% pirenzepine was associated with similar effects in reducing the progression of myopic refractive error (*P* = 0.016 and *P* = 0.0006, respectively) and axial elongation (*P* = 0.017and *P* = 0.0004, respectively). In a study performed by Carr and colleagues, intravitreally injected brimonidine (20 nmol and 200 nmol) and clonidine (200 nmol) inhibited experimentally induced increases in myopic refractive error and axial elongation in white Leghorn chicks with form deprivation myopia [22]. While IOP was not measured in Carr’s study, the authors concluded that high concentrations of α2-adrenoceptor agonists inhibited form deprivation myopia in chicks.

The discrepancy in the results between El-Nimri’s study and Liu’s study as well as our investigation may have been due to differences in the IOP-lowering agent (latanoprost versus brimonidine versus carteolol), including differences in the duration of the IOP-lowering effect. In humans, the IOP-lowering effect lasts for approximately 24 h with prostaglandin derivatives such as latanoprost, while the ocular hypotensive effect of beta-blockers is mostly limited to daytime hours [26].

The IOP measurements reported in our study (15.0 ± 3.0 mmHg at the age of 2 weeks at baseline; 15.5 ± 3.5 mm in the left control eyes at study end) were slightly higher than those reported in some previous investigations. In the study by Cairó and colleagues, guinea pigs aged 4 weeks and guinea pigs aged 3 to 36 months showed tonometric measurements of 8.53 ± 1.28 mmHg and 13.20 ± 1.28 mmHg, respectively, as measured by a rebound tonometry between 4 pm and 6 pm [27]. Applanation tonometry revealed values of 10.9 ± 3.6 mmHg for the adult group. In the investigation performed by Rajaei and associates, IOP was measured by rebound tonometry (TonoVet tonometer, iCare, Tiolat, Helsinki, Finland) in guinea pigs aged 12 to 15 months and was 6.81 ± 1.41 mmHg [28]. The IOP measurements obtained in our study by applanation tonometry were considerably lower than the IOP readings obtained by rebound tonometry in the recent study by El-Nimri, with mean values ranging between 22.2 ± 1.0 mmHg and 24.2 ± 0.09 mmHg in the various subgroups at baseline (Table 4).

Interestingly, the idea that IOP does not promote axial elongation is supported by observations in several different animal models, in which sectorial form deprivation or sectorial negative defocus induced sectorial axial myopia, although the IOP was equal in all regions of the eyes. It is also supported by the clinical finding that a marked increase in IOP does not lead to excessive elongation in emmetropic eyes and the normalization of an elevated IOP does not lead to the shortening of the axial length.

Limitations of our study should be discussed. First, we looked for associations between IOP and axial elongation. Even if we had found a statistically significant correlation, we would not have been able to conclude that the reduction in IOP caused the reduction in axial elongation since the reverse of such a causal relationship would also have been possible. Such a limitation may also be valid for previous studies. Similarly, the higher IOP that was observed in individuals with a longer axial length, as measured in hospital-based studies as well as in population-based investigations, might have been a secondary effect of axial elongation on the anterior segment. Second, we did not record calibration measurements for the tonometer we used in our study. Third, we did not assess the effect of carteolol in normal eyes without lens-induced myopization. Fourth, the differences in axial length and axial elongation were relatively small, so the statistical power of the study might not have been sufficient to reveal a statistically significant association. Fifth, we used a Tono-Pen device for tonometry, while in some studies, devices for rebound tonometry have shown better performance (Table 4) [29]. Sixth, the results of this study may be specific to carteolol rather than beta blockers in general since the group of beta blockers with substances such as carteolol and timolol differ in their pharmacodynamics and receptor specificity. The negative result regarding the use of carteolol may therefore not lead to generalized conclusions for other IOP lowering drugs including other beta blockers or prostaglandins. Seventh, the sample size in this study was relatively small, so the lack of statistical significance in the results might have been caused by a limited amount of statistical power regarding the study sample. Eighth, we used topically applied carteolol to lower the IOP and to determine whether an association exists between IOP and axial elongation. To test Koch’s hypotheses, future studies should be conducted to determine the effect of an elevated IOP on the ocular axial length.

## Conclusions

In young guinea pigs with or without lens-induced axial elongation, neither the physiological IOP nor the IOP reduced by a topical beta-blocker, carteolol, was significantly associated with the magnitude of axial elongation. These results suggest that IOP, regardless of whether it is influenced by carteolol, does not play a major role in axial elongation in young guinea pigs.

## Data Availability

The original data of the current study are available from the corresponding author upon reasonable request.

## Abbreviations

IOP: Intraocular pressure
RE: Refractive error
D: Diopter
AL: Axial length
CI: Confidence interval
Fig: Figure
